# Our Daughters

**Published:** 1856-11

**Authors:** 


### OUR DAUGHTERS

ARE the hope of our country's future. Their physical, moral and domestic education, are of an importance which no array of figures can express, which multitudes of ponderous tomes could not adequately portray.

As is the mother, so is the man. If she be a woman of physical vigor, a high guaranty is given of healthy children. If her moral character is pure, formed in the mould of Bible piety, we may anticipate for her offspring, lives of the self same piety, with its benevolent influences spreading far and wide, from all their habitations.

If the mother in her domestic relations, be a pattern for all that is cleanly and systematic, and punctual and prompt and persevering, with womanly dignity and lovingness pervading all, then may we look for every son of such a woman to be a man of mark for his time, and for every daughter, to become a wife well worthy of a king.

When such destinies hang upon the future of our daughters, ought they to be hurried from a loving mother's side at seventeen, at fifteen, at twelve, to the purchased care of a governess? To the herded tuition of fashionable boarding schools, where glitter and superficiality and empty show predominate; where nothing that is radically useful and good is thorough; where associations are inevitable, with the children of the parvenue, as well as with the scion of the decayed aristocrat, thus exposing the pure heart to the withering and corrupting examples of mere pretence and of baseless pride?

The theatre, the ball-room, the sea-shore, or the Spa—are these the schools to mould aright the character of the girls who are to be the mothers of the next generation? Is the heterogeneous weekly newspaper, the trashy monthly, the "last novel," be it from whom it may—are these suitable text books to form the principles of her who is so soon to become the wife, the mother, the matron?

We trust these suggestive inquiries will arrest the attention and command the mature reflection of every parent who reads this article.

